# Novel and Lost Forests in the Upper Midwestern United States, from New Estimates of Settlement-Era Composition, Stem Density, and Biomass

**DOI:** 10.1371/journal.pone.0151935

**Published:** 2016-12-09

**Authors:** Simon J. Goring, David J. Mladenoff, Charles V. Cogbill, Sydne Record, Christopher J. Paciorek, Stephen T. Jackson, Michael C. Dietze, Andria Dawson, Jaclyn Hatala Matthes, Jason S. McLachlan, John W. Williams

**Affiliations:** 1 Department of Geography, University of Wisconsin-Madison, Madison, Wisconsin, United States; 2 Department of Forest and Wildlife Ecology, University of Wisconsin-Madison, Madison, Wisconsin, United States; 3 Harvard Forest, Harvard University, Petersham, Massachusetts, United States; 4 Harvard Forest, Harvard University, Petersham, Massachusetts, United States; 5 Department of Biology, Bryn Mawr College, Bryn Mawr, Pennsylvania, United States; 6 Department of Statistics, University of California, Berkeley, California, United States; 7 Department of the Interior Southwest Climate Science Center, U.S. Geological Survey, Tucson, Arizona; 8 School of Natural Resources and the Environment and Department of Geosciences, University of Arizona, Tucson, Arizona, United States; 9 Department of Earth and Environment, Boston University, Boston, Massachusetts, United States; 10 Department of Statistics, University of California, Berkeley, California, United States; 11 Department of Geography, Dartmouth College, Hanover, New Hampshire, United States; 12 Department of Biological Sciences, University of Notre Dame, Notre Dame, Indiana, United States; 13 Department of Geography, University of Wisconsin-Madison, Madison, Wisconsin, United States; 14 Center for Climatic Research, University of Wisconsin-Madison, Madison, Wisconsin, United States; Chinese Academy of Forestry, CHINA

## Abstract

**Background:**

EuroAmerican land-use and its legacies have transformed forest structure and composition across the United States (US). More accurate reconstructions of historical states are critical to understanding the processes governing past, current, and future forest dynamics. Here we present new gridded (8x8km) reconstructions of pre-settlement (1800s) forest composition and structure from the upper Midwestern US (Minnesota, Wisconsin, and most of Michigan), using 19th Century Public Land Survey System (PLSS), with estimates of relative composition, above-ground biomass, stem density, and basal area for 28 tree types. This mapping is more robust than past efforts, using spatially varying correction factors to accommodate sampling design, azimuthal censoring, and biases in tree selection.

**Changes in Forest Structure:**

We compare pre-settlement to modern forests using US Forest Service Forest Inventory and Analysis (FIA) data to show the prevalence of lost forests (pre-settlement forests with no current analog), and novel forests (modern forests with no past analogs). Differences between pre-settlement and modern forests are spatially structured owing to differences in land-use impacts and accompanying ecological responses. Modern forests are more homogeneous, and ecotonal gradients are more diffuse today than in the past. Novel forest assemblages represent 28% of all FIA cells, and 28% of pre-settlement forests no longer exist in a modern context. Lost forests include tamarack forests in northeastern Minnesota, hemlock and cedar dominated forests in north-central Wisconsin and along the Upper Peninsula of Michigan, and elm, oak, basswood and ironwood forests along the forest-prairie boundary in south central Minnesota and eastern Wisconsin. Novel FIA forest assemblages are distributed evenly across the region, but novelty shows a strong relationship to spatial distance from remnant forests in the upper Midwest, with novelty predicted at between 20 to 60km from remnants, depending on historical forest type. The spatial relationships between remnant and novel forests, shifts in ecotone structure and the loss of historic forest types point to significant challenges for land managers if landscape restoration is a priority. The spatial signals of novelty and ecological change also point to potential challenges in using modern spatial distributions of species and communities and their relationship to underlying geophysical and climatic attributes in understanding potential responses to changing climate. The signal of human settlement on modern forests is broad, spatially varying and acts to homogenize modern forests relative to their historic counterparts, with significant implications for future management.

## Introduction

Composition, demography, and structure of forests in eastern North America has changed continuously over the last millennium, driven by changes in human land-use [1–5] and climate variability [6–9]. While human effects have been a component of these systems for millennia, the EuroAmerican settlement and industrialization period has increased anthropogenic effects by orders of magnitude [10–12]. Legacies of post-settlement land-use in the upper Midwest [13] and elsewhere have been shown to persist at local and regional scales [5,14,15], and nearly all North American forests have been affected by the intensification of land-use in the past three centuries. Hence, contemporary ecological processes in North American forests integrate the contemporary and historical anthropogenic impacts of the EuroAmerican settlement period and natural influences at decadal to centennial scales.

Multiple major ecotones exist within the upper Midwestern United States (US), including the prairie-forest boundary, historic savanna, and the Tension Zone between southern and northern forests [16]. Large and well-documented changes in forest structure and composition have occurred in this region since EuroAmerican settlement [13,17–20]. The extent to which ecotones have shifted, and their extent both prior to and following EuroAmerican settlement is of critical importance to biogeochemical and biogeophysical vegetation-atmosphere feedbacks [21], carbon sequestration [17], and regional management and conservation policy [22–25].

At a regional scale many modern forests in the upper Midwest (*i*.*e*., Minnesota, Wisconsin and Michigan) have low species richness and functional diversity relative to forests of the pre-EuroAmerican settlement (hereafter pre-settlement, *ca*. mid-late 1800s) period [26–28] due to near-complete logging, often followed by severe wildfire. For example, forests in Wisconsin are in a state of regrowth, with an unfilled carbon sequestration potential of 69 TgC [17] as a consequence of land cover conversion and subsequent recovery following abandonment of farm lands in the 1930s. Differences in land-use history across the Midwest, superimposed on original vegetation patterns and modern environmental gradients, may have led, not only to broad spatial patterns, but also to significant local-to-regional variation. For example, while fire suppression occurred throughout the region, effects of suppression have and will continue to manifest themselves differently depending on the historical vegetation and biophysical characteristics of the site or region.

Land-use legacies emerge at regional scales [29–31]. Under intensive land-use change, natural processes of succession, senescence and recruitment may be heavily altered or redirected. Broad-scale land-use change can drive changes in forest structure, species pools, and ecosystem properties that may not be apparent on the relatively narrow time scales at which ecological processes are traditionally observed [30,32]; modern chronosequences may miss important changes in structure and composition. The recolonization of forested landscapes following agricultural clearance in the upper Midwest [20], highlights the importance of understanding ecological trajectories and land-use legacies in understanding modern forest dynamics [29]. Cramer *el al*. [33] cite successional theory to suggest that many old fields will return to a 'natural' state, but point out that recovery is not universal. In particular, intense fragmentation of the landscape can deplete the regional species pool, leading to failures of recruitment that might favor species with longer distance seed dispersal [34].

While debate persists over how to conceptualize and identify novel ecosystems, and their scientific and management implications [35–37], the fact remains that there are now forest and vegetation communities on the landscape without historic analogues that must be managed [38]. Long-term management of forest ecosystems and their associated services requires understanding the extent to which landscapes have been modified by historic land-use and the spatial (and temporal) scales at which novel ecosystems arise. While restoration efforts have generally focused on ecosystems at local scales, there is an increasing need to focus on management and restoration at landscape scales [39]. An understanding of landscape-level processes driving ecological novelty can help prioritize intervention strategies at local scales [40], and provide a better understanding of the role of remnant patches in restoring hybrid or novel landscapes.

Building upon prior work [18,26,27,27,41–47] and the United States Forest Service—Northern Central Research Station (http://www.ncrs.fs.fed.us/gla/), we use the Public Land Survey System (PLSS) to derive estimates of pre-settlement (*ca*. mid-late 1800s) forest composition, basal area, stem density, and biomass. Most prior PLS-based reconstructions are for individual states or smaller extents [17,18,27,31,48] often aggregated at the scale of regional forest zones [26,27], although aggregation may also occur at the section [17] or township scale [49]. With spatially extensive historical datasets for the Upper Midwestern United States it becomes possible to quantify the extent of change in forest structure and composition since EuroAmerican Settlement. There is a critical need for a rigorously vetted, and quantitatively robust mapping of historical forest communities that is compatible with modern forest cover data such as the Forest Inventory and Analysis data produced by the US Forest Service. Forest cover data at two time points, separated by major land use conversion can provide greater insight into forest structure and function than any single dataset, and, further, will allow us to cross time-scales, for example, with the use of pollen data [50].

Forest Inventory and Analysis (FIA) forest surveys, which began in the 1930s, are the closest modern equivalent of the spatially extensive PLSS data. Modern forest structure and composition data [51] play a ubiquitous role in forest management, conservation, carbon accounting, and basic research on forest ecosystems and community dynamics. In general, FIA datasets are systematically organized and widely available to the forest ecology and modelling community [52], whereas most PLSS data compilations are of local, or at most, state-level extents. This absence of widely available data on settlement-era forest composition and structure has been a major barrier to understanding and modeling the current and future processes governing forest dynamics at broader, regional scales. For example, distributional models of tree species often rely upon FIA or other contemporary observational data to build species-climate relationships that can be used to predict potential range shifts [53,54], without consideration of historical forest data.

Our work develops new approaches to address well known and substantial challenges to the ecological interpretation and application of PLSS data, including lack of standardization in tree species names [55], azimuthal censoring by surveyors [41], variations in sampling design over time [56], and differential biases in tree selection among different kinds of survey points within the survey design at any point in time [42,57]. The correction factors developed here are spatially varying, allowing us to accommodate temporal and spatial variations in surveyor methods.

We aggregate point-based estimates of stem density, basal area and biomass to an 8 x 8km grid, and classify forest types in the upper Midwest to facilitate comparisons between FIA and PLSS data. We compare the PLSS data to late-20th-century estimates of forest composition, tree stem density, and basal area. Using analog analyses, we identify lost forests with no close compositional counterpart today, and novel forests with no close historical analogs, and we model the spatial relationships between cells with novel forest types and those that had close historical counterparts. We explore how forest homogenization has changed the structure of two major ecotones from southern deciduous to northern evergreen forests, and the forest-prairie boundary. This work provides insight into the compositional and structural changes between historic and contemporary forests, while setting the methodological foundation for a new generation of regional to subcontinental-scale maps and analyses of settlement-era forests in the Eastern US.

## Methods

### Public Lands Survey Data: Assembly, and Standardization

The PLSS was designed to facilitate the division and sale of federal lands from Ohio westward and south. The survey created a 1 mile^2^ (2.56 km^2^) grid (sections) on the landscape. At each section corner and half-mile (quarter-section) point, a stake was placed as the official location marker. To mark these survey points, PLSS surveyors recorded tree stem diameters, measured distances and azimuths of the two to four trees 'closest' to the survey point and identified tree taxa using common (and often regionally idiosyncratic) names. PLSS data thus represent measurements by hundreds of surveyors from 1832 until 1907, with changing sets of instructions over time [56,58]. The work presented here builds upon prior digitization and classification of PLSS data for Wisconsin [46,47], Minnesota [43] and Michigan (USFS-NCRS).

The PLSS replaced earlier Town Proprietor Surveys (TPS) used in the northeastern US and British Colonies [2,59]. The TPS provided estimates of relative forest composition at the township level, but no structural attributes. The PLSS produced spatially explicit point level data, with information about tree spacing and diameter, which can be used to estimate absolute tree density and biomass. PLSS notes include tree identification at the point level, disturbance [60] and other features of the pre-settlement landscape. However, uncertainties exist within the PLSS [61].

Ecological uncertainty in the PLSS arises from the dispersed spatial sampling design (fixed sampling every half mile), precision and accuracy in converting surveyor's use of common names for tree species to scientific nomenclature [55], digitization of the original survey notes, and surveyor bias during sampling [42,61–63]. Estimates vary regarding the ecological significance of surveyor bias. Terrail *et al*. [64] show strong fidelity between taxon abundance in early land surveys versus old growth plot surveys. Liu *et al* [42] estimate the ecological significance of some of the underlying sources of bias in the PLSS and show ecologically significant (>10% difference between classes) bias in species and size selection for corner trees. However Liu *et al*. [42] also indicate that the true sampling error cannot be determined, particularly because most of these historic ecosystems are largely lost to us.

Kronenfeld and Wang [41], working with historical witness tree datasets in western New York, indicate that direct estimates of density using plotless estimators may be off by nearly 37% due to azimuthal censoring (*i*.*e*., the tendency of surveyors to avoid trees close to cardinal directions), while species composition estimates may be adjusted by between -4 to +6%, varying by taxon, although Kronenfeld [57] shows adjustments of less than 1%. These biases can be minimized by appropriate analytical decisions; many efforts over the years have assessed and corrected for the biases and idiosyncrasies in the original surveyor data [27,41,42,63,65–69]. Even given these caveats, PLSS records remain the best source of data about both forest composition and structure in the United States prior to EuroAmerican settlement.

**Fig 1:**
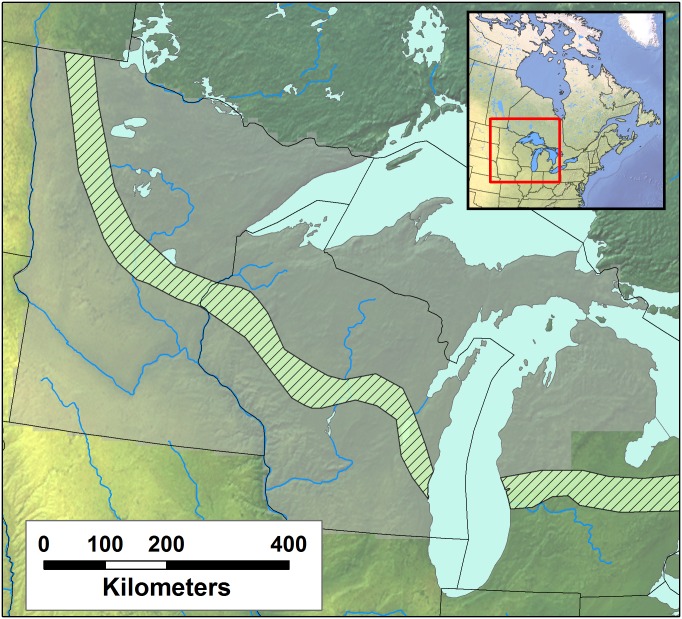
The domain of the Public Land Survey investigated in this study. The broad domain includes Minnesota, Wisconsin and the upper two thirds of Michigan state (greyed cells). A 8x8km grid is superimposed over the region to aggregate data, resulting in a total of 7940 cells containing data. The striped band represents the Tension Zone [16], adapted across the region from Andersen [72].

This analysis builds upon and merges previous state-level efforts to digitize and database the point-level PLSS data for Wisconsin, Minnesota and the Upper Peninsula and upper third of the Lower Peninsula of Michigan. These datasets were combined using spatial tools in R [70,71] to form a common dataset for the upper Midwest (Fig 1) using the Albers Great Lakes and St Lawrence projection (see code in S1 File: *step_one_clean_bind*.*R*; proj4: *+init*:*EPSG*:*3175*).

We took several steps to standardize the dataset and minimize the potential effects of surveyor bias upon estimates of forest composition, density, and biomass. All steps are preserved in the supplementary R code (S1 File: *step_one_clean_bind*.*R*). We excluded line and meander trees (i.e. trees encountered along survey lines, versus trees located at section or quarter-section corners). Surveyor selection biases for tree size and species appear to have been more strongly expressed for line trees. Meander trees were used to avoid obstacles, such as water-bodies, and so have non-random habitat preferences [42]. Lastly, there are inherent differences in sampling design between line, meander and corner points. We used only the closest two trees at each corner point because the third and fourth farthest trees have stronger biases with respect to species composition and diameter [42]. Corner points were used only if 1) there were at least two trees (or non-tree records, *e*.*g*. "rock" or "No Tree"), 2) the two trees were from different quadrants (defined by the cardinal directions), and 3) there were valid azimuths to the trees (a defined quadrant with an angle between 0 and 90) and valid diameters (numeric, non-zero).

Many species-level identifications used by PLSS surveyors are ambiguous. Statistical models can predict the identity of ambiguous species [55], but these models introduce a second layer of uncertainty into the compositional data, both from the initial surveyors' identification, and from the statistical disambiguation. Given the regional scale of the analysis, and the inherent uncertainty in the survey data itself, we chose to avoid this layer of taxonomic uncertainty, and retained only genus-level identification (S2 File). The ecological implications for the use of genera-level taxonomies are important for this region. While fire tolerance is fairly well conserved within genera, shade tolerance can vary. *Betula* contains shade intolerant *B*. *papyrifera* and the intermediate *B*. *alleghaniensis*, while *Pinus* contains the very shade intolerant *P*. *banksiana*, the intolerant *P*. *resinosa* and the moderately tolerant *P*. *strobus*. For cases where shade tolerance (or fire tolerance) varies strongly within a genera we examine the data to determine the suitability of the assignment, or extent of confusion within the assigned genera.

In areas of open prairie or other treeless areas, *e*.*g*. southwestern Minnesota, surveyors recorded distances and bearings to 'Non Tree' objects. When points were to be located in water bodies the point data indicates 'Water'. Points recorded 'No Tree' are considered to have been from extremely open vegetation, with an estimated point-level stem density of 0 stems ha^-1^. We based our estimates on terrestrial coverage, so water cells are excluded completely. Hence, absence of trees at 'No Tree' locations does reduce the gridded estimates of terrestrial stem density, but absence of trees at 'Water' locations does not.

Digitization of the original surveyor notebooks introduces the possibility of transcription errors. The Wisconsin dataset was compiled by the Mladenoff lab group, and has undergone several revisions over the last two decades in an effort to provide accurate data [22,42,46,47,55]. The Minnesota transcription error rate is likely between 1 and 5%, and the treatment of azimuths to trees varies across the dataset [43]. Michigan surveyor observations were transcribed to Mylar sheets overlaid on State Quadrangle maps, so that the points were displayed geographically, and then digitized to a point based shapefile (Ed Schools, pers. comm.; Great Lakes Ecological Assessment. USDA Forest Service Northern Research Station. Rhinelander, WI. http://www.ncrs.fs.fed.us/gla/), carrying two potential sources of transcription error. Preliminary assessment of Southern Michigan data indicates a transcription error rate of 3–6%. To reduce errors associated with transcription across all datasets, we exclude sites for which multiple large trees have a distance of 1 link (20.12 cm) to point center, trees with very large diameters (diameter at breast height—dbh > 100 in; 254 cm), points where the azimuth to the tree is unclear, and points where the tree is at point center but has a recorded azimuth. All removed points are documented in the code used for analysis (S1 File: *step_one_clean_bind*.*R*) and are commented for review.

### Data Aggregation

We binned the point data using a 64km^2^ grid (Albers Gt. Lakes St Lawrence projection; S1 File: *base_calculations*.*R*) to create a dataset that has sufficient numerical power for spatial statistical modeling and sufficient resolution for regional scale analysis [73]. This resolution is finer than the 100km^2^ gridded scale used in Freidman and Reich [18], but coarser than township grids used in other studies [17,57] to provide a scale comparable to aggregated FIA data at a broader scale. Forest compositional data is based on the number of individuals of each genus or plant functional type (PFT) present at all points within a cell. Stem density, basal area and biomass are averaged across all trees at all points within the cell.

### Stem Density

Estimating stem density from PLSS data is based on a plotless density estimator that uses the measured distances from each survey point to the nearest trees at the point location, the Morisita density estimator [74,75]. The Morisita density estimate is then corrected to minimize error due to different sampling geometries and several known surveyor biases [27,41,42,63,65,67–69]. The standardized approach for this method is well validated, however surveyors did not use a consistent approach to point level sampling. Survey sampling instructions changed throughout the implementation of the PLSS in this region and differed between section and quarter-section points and between internal and external points within a township [42,56]. Our approach allows for spatial variation in surveyor methods by applying correction factors based on the empirical sample geometry, and known surveyor biases deviating from this design (Cogbill, *pers*. *comm*.). These estimates are based on empirical examination of the underlying data, and have been validated using simulations on stem mapped stands (Cogbill, *pers*. *comm*.).

We estimate stem density (stems m^-2^) based on the Morisita two-tree density estimator, which uses the distance-to-tree measurements for the two closest trees at each point [76]. The correction to the estimate uses explicit and spatially varying factors that account for variations in sampling designs over time and among surveyors. All code to perform the analysis is included in S1 File (*misc*.*functionsv1*.*4*.*R*).

We estimate the basic stem density (stems m^-2^) using the point-to-tree distances for the closest trees to each point within a defined number of sectors around the point [74]:

$$\lambda_{\hat{m_{2}}}=\frac{k-1}{\pi \times n}\times \sum_{i=1}^{N}\frac{k}{\sum_{j=1}^{k}{\left(r_{ij}\right)}^{2}}$$

where *λ* is density; *k* is the number of sectors within which trees are sampled, *N* is the number of points over which estimates are aggregated, *r* is the distance of point-to-tree (as m). This estimate can be modified [76,77], which creates a correction, herein called *κ*, that accounts for different sampling designs. This "Cottam" correction factor recognizes that different sampling designs, which affect the number and order of the distances in different quadrants (or sectors), will lead to different apparent tree densities. When either four quadrants or trees are sampled (point quarter design), or when two trees in opposite semicircles (point halves design) are sampled, the equation is accurate and *κ* = 1; when the two trees are in the nearest of two quadrants (two nearest quadrants design), *κ* = 0.857; and when two trees are in quadrants on the same side of the direction of travel (one-sided or interior half design), *κ* = 2. This parameter, in Cottam's notation [77], is a divisor of the denominator above, or here, the mathematically equivalent multiplier in the numerator of the reciprocal of the squared distances.

We further simplify the density equation so that it is calculated at each point (N = 1) and for two sample trees only (k = 2):

$$\lambda_{M}=\frac{2}{\pi \times \sum_{j=1}^{2}r_{j}{}^{2}}$$

Then the point values for any sampling design can be Cottam corrected (*κ* × *λ*_*M*_). For example, the basic Morisita equation for two sectors assumes trees are located in opposite halves, so if the actual design is the nearest tree in the two nearest quadrants, the density from the simplified equation will be overestimated and must be correspondingly corrected by multiplying by *κ* = 0.857.

Further corrections account for the restriction of trees to less than the full sector (*θ*), censoring of trees near the cardinal azimuths (*ζ*), and under-sampling of trees smaller than a certain diameter limit (*ϕ*). These parameters are derived from analyses of measurements of bearing angles and diameters actually observed in surveys of witness trees within a subset of townships across the upper Midwest.

Sector bias (*θ*). Although the density model for two tree points assumes that the trees are on opposite sides of a sample line (point halves), the actual sample is often more restricted (< 180°) within the sector, or is a less restricted (> 180°) angle beyond the sector (see S3 File). This deviation from the equation's assumption of equal distribution of angles across the 180° sector is quantified using the empirical angle between the bearings of the two trees (pair angle). The pair angle frequencies (S3 File) that the observed proportion of trees (p) within any restricted sector divided by the proportion of that angle within the circle (*α*) are an estimate of the bias imposed by the actual sampling [41]. The factor (*θ* = p/*α*) indicates bias associated with differences in geometry of two tree samples. This parameter (*θ*) varies from 0.71 to 1.27, indicating sampling from effectively 253° to 141° sectors.

Azimuthal censoring (*ζ*). In addition to sector bias, surveyors did not always sample trees near the cardinal directions [41,67,68]. This azimuthal censoring is commonly found along the line of travel on section lines and sometimes on the perpendicular quarter-section lines. Trees near the cardinal directions were passed over, and a replacement was found within a more restricted angular region. The correction for this bias is calculated following Kronenfeld and Wang [41] in a manner similar to the sector bias. The factor *ζ* is the ratio of the proportion of trees in the restricted area (p) divided by the proportion of the complete circle (*α*) that is used. The azimuthal censoring parameter (*ζ*) ranges from 1.03 to 1.25 indicating an equivalent to complete elimination of trees from 10° to 72° azimuths adjacent to the cardinal directions.

Diameter limit (*ϕ*). Examination of the diameter distributions from settlement era surveys across the upper Midwest clearly demonstrate witness trees less than 8 inches (20 cm) in diameter were under-sampled [42,66,68]. We have confirmed this bias in our own inspection of plots of diameter frequency in the PLSS data, which show a sharp drop in the frequency of reported diameters below 8". This bias can be accommodated by setting a diameter limit, and only calculating the density for trees with diameters above this limit. Total density calculated from all trees is reduced to this reference limit by simply multiplying the total by the percentage of trees above this limit. This effectively eliminates the smaller trees from the total and normalizes the value of trees above this standard. The parameter (*ϕ*) represents diameter size bias is simply the percentage of trees ≥ 8" and, in practice, ranges from 0.6–0.95.

If all surveyor bias corrections are independent and do not overlap, they are simple multipliers of the model density, and the bias-minimized estimate of the point density of trees ≥ 8" (20 cm) is:

$$\lambda_{Mcorrected}=\kappa \times \theta \times \zeta \times \phi \times \lambda_{M}$$

Estimates for each point *i* can be averaged for all *N* points in any region. Correction factors are calculated separately for different regions, years, internal versus external lines, section versus quarter-section points, and surveyor sampling designs (S4 File). All code to perform the analyses is included in S1 File. Simulations using stem mapped stands from the region (Cogbill, *pers comm*) supports the robustness of this method, as opposed to other methods presented in the literature.

### Basal Area and Biomass Estimates

Forest basal area is calculated by multiplying the point-based stem density estimate by the average stem basal area from the reported diameters at breast height for the closest two trees at the point (n = 2). Aboveground dry biomass (Mg ha^-1^) is calculated using the USFS FIA tree volume and dry aboveground biomass allometry equations for the United States [78].

Biomass equations share the basic form:

$$m=Exp\left(\beta_{0}+\beta_{1}*\text{ln}dbh\right)$$

where *m* represents stem biomass for an individual tree in kg. *β*_0_ and *β*_1_ are parameters derived from [78] and described in Table 1. *dbh* is the stem diameter at breast height (converted to cm) recorded in the survey notes. The biomass estimates are averaged across both trees at a survey point and multiplied by the stem density calculated at that point to produce an estimate of aboveground biomass reported in Mg ha^-1^ [78].

**Table 1:** Biomass parameters used for the calculation of biomass in the pre-settlement dataset (rounded for clarity).

Jenkins Species Group	*β*_0_	*β*_1_	PalEON Taxa Included (Supp. 2)
Aspen, Alder, Poplar, Willow	-2.20	2.38	Poplar, Willow, Alder
Soft Maple, Birch	-1.91	2.36	Birch
Mixed Hardwood	-2.48	2.48	Ash, Elm, Maple, Basswood, Ironwood, Walnut, Hackberry, Cherries, Dogwood, Buckeye
Hard Maple, Oak, Hickory, Beech	-2.01	2.43	Oak, Hickory, Beech, Other Hardwood
Cedar and Larch	-2.03	2.26	Tamarack, Cedar
Fir and Hemlock	-2.54	2.43	Fir, Hemlock
Pine	-2.54	2.43	Pine
Spruce	-2.08	2.33	Spruce

Matching PLSS tree genera to the species groups defined by Jenkins *et al*. [78] is straightforward, placing the 28 genera used in this study into 8 allometric groups (Table 1). However, all maples are assigned to the generic "Hardwood" group since separate allometric relationships exist for soft and hard maple (Table 1). Biomass estimates for "Non tree" survey points are assigned 0 Mg ha^-1^.

We use the stem density thresholds of Anderson and Anderson [44] to discriminate prairie, savanna, and forest.

### FIA Stem Density, and Basal Area

The United States Forest Service has monitored the nation's forests through the FIA Program since 1929, with an annualized state inventory system implemented in 1998 [52]. On average there is one permanent FIA plot per 2,428 ha of land in the United States classified as forested. Each FIA plot consists of four 7.2m fixed-radius subplots in which measurements are made of all trees >12.7cm dbh [52]. We used data from the most recent full plot inventory (2007–2011). The FIA plot inventory provides a median of 3 FIA plots per cell using the 64km^2^ grid. We calculated mean basal area (m^2^ ha^-1^), stem density (stems ha^-1^), and mean diameter at breast height (cm) for all live trees with dbh greater than 20.32cm (8in). All calculations followed instructions in Woudenberg *et al* [52].

One critical issue is the reliance on forested condition for the FIA sampling. This reduces our capacity to compare forest state between PLSS and FIA cover in regions with historical prairie and savanna coverage that are now patches of closed canopy forest among a largely agricultural landscape. In addition, it may result in the overestimation of modern density and basal area at the mesoscale in these same regions by drawing from a sample biased specifically towards plots with > 10% forest cover [52], however, the 10% cover threshold is fairly low, but more likely in line with "open forest" [44] than savanna.

### Gridding and Analysing PLSS and FIA Data

Maps of stem density, basal area and biomass (for PLSS) were generated by averaging all PLSS point or FIA plot estimates within a 64km^2^ raster cell. Differences in sampling design between PLSS and FIA data combined with spatially structured forest heterogeneity will affect the partitioning of within-cell versus between-cell variance, but not the expected estimates. Most 64km^2^ cells have one or a few intensively sampled FIA plots. Therefore at this scale of aggregation, the low density of FIA plots in heterogeneous forests could result in high within-cell variance and high between-cell variability. For the PLSS plotless (point based) estimates, stem density estimates are sensitive to trees close to the corner. Point-level estimates with very high stem densities can skew the rasterized values, and it is difficult to distinguish artifacts from locations truly characterized by high densities. To accommodate points with exceptionally high densities we carry all values through the analysis, but exclude the top 2.5 percentile when reporting means and standard deviations in our analysis. PLSS-based estimates are highly variable among points due to the small sample size, but have low variance among 64 km^2^ raster cells due to the uniform sampling pattern of the data. Thus, within-cell variance is expected to be high for the PLSS point data, but spatial patterns are expected to be robust at the cell level. The base raster and all rasterized data are available as S3 File.

Standard statistical analysis of the gridded data, including correlations, paired t-tests and regression, was carried out in R [70], and is documented in supplementary material that includes a subset of the raw data to allow reproducibility. Analysis and presentation uses elements from the following R packages: cluster [79], ggplot2 [80,81], gridExtra [82], igraph [83], mgcv [84], plyr [85], raster [86], reshape2 [87], rgdal [71], rgeos [88], sp [89,90], statmod [91], and spdep [92].

We identify analogs and examine differences in composition between and within PLSS and FIA datasets using Bray-Curtis dissimilarity [93] for proportional composition within raster cells using the relative basal area of each species. For the analog analysis we are interested only in the minimum compositional distance between a focal cell and its nearest compositional (not spatial) neighbor. The distribution of compositional dissimilarities within datasets indicates forest heterogeneity within each time period, while the search for closest analogs between datasets indicates whether contemporary forests lack analogs in pre-settlement forests ('novel forests'), or vice versa ('lost forests'). For the analog analyses, we compute Bray-Curtis distance between each 64km^2^ cell in either the FIA or the PLSS periods to all other cells within the other dataset (FIA to FIA, PLSS to PLSS), and between datasets (PLSS to FIA and FIA to PLSS), retaining only the minimum. For within era analyses (FIA to FIA and PLSS to PLSS), cells were not allowed to match themselves. We define vegetation classes for lost and novel forests using k-medoid clustering [79].

The differences in sampling design and scale between the PLSS and FIA datasets, described above, potentially affect between-era assessments of compositional similarity [49]. The effects of differences in scale should be strongest in regions where there are few FIA plots per 64 km^2^ cell, or where within-cell heterogeneity is high. For the analog analyses, this effect should increase the compositional differences between the FIA and PLSS datasets.

Because Bray-Curtis dissimilarity is sensitive both to species turnover and to species richness, we expect that more FIA plots within a cell should result in greater richness (due to sampling effects) and thus, greater turnover between cells. We test for the importance of this effect on our analog analyses via a sensitivity analysis in which we test whether dissimilarities between FIA and PLSS grid cells are affected by the number of FIA plots per cell. In this region richness, dissimilarity and FIA plot number are all collinear because of a regional collinearity in species richness that exists in spite of sampling effect. Because of this colinearity we limit our analysis to a simple test for cell number effect since it becomes increasingly difficult to partition the effects of the shift from monotypic oak savanna, which has low forest cover, and thus low FIA sampling intensity, to the diverse mixedwood forests of north-central Wisconsin. We do find a small but significant effect (see below), suggesting that our analyses are mainly sensitive to the compositional and structural processes operating on large spatial scales.

To understand the extent to which the processes governing novelty operate at landscape scales, we relate the novelty of a cell to the spatial distance between individual novel cells and the nearest 'remnant' forest cell, *i*.*e*., what is the minimum distance from a remnant forest cell at which all cells are predicted to be novel. Novel forests are defined as any cell in the FIA dataset for which dissimilarity to the PLSS era is above the 95%ile of dissimilarities within the PLSS data. We examine whether the distance to novel forest relationship varies between forest types, and whether it is different than the relationship we might see if the dissimilarity values were distributed randomly on the landscape. The definition of "remnant" forest is likely to be arbitrary and, possibly, contentious. We use a threshold, the lowest 25%ile of compositional dissimilarity within the PLSS data, as our cutoff. This means that all FIA cells with nearest neighbor dissimilarities to the PLSS era forests below this cutoff are considered to be representative of the PLSS era forests. The analysis presented below is robust to higher cutoffs for the remnant forest threshold.

We use a generalized linear model with a binomial family to relate novelty (as a binomial, either novel or not) to the spatial distance from the nearest 'remnant' cell for each of the five major forest types within the PLSS data (Oak savanna, Oak-Poplar-Basswood-Maple, Pine, Hemlock-Cedar-Birch-Maple and Tamarack-Pine-Spruce-Poplar forests). Because the geographic extent of this region is complex, with islands, peninsulas and political boundaries, we use permutation, resampling the FIA to PLSS nearest neighbor distances without replacement, to estimate the expected distance to novelty if FIA to PLSS nearest neighbor dissimilarities were distributed randomly on the landscape.

We expect a weak relationship will indicate that novelty, following landscape-scale land-use change, is moderated by a species pool culled from small remnant patches, individual specimens, or local scale restoration efforts. A significant relationship between distance from remnant forest and novelty indicates that small patches have been insufficient to restore natural forest cover within the region, and would indicate that greater efforts are needed to restore landscapes at regional scales.

To illustrate the spatial effects of land use changes on forest composition, we select two linear transects through the region and model vegetation using smooth splines as part of a generalized additive model [84] using R [70]. This smoothing is used for visualization purposes only, but is applied to each individual taxon using the beta regression family ("*betar*"). We use smoothing to reduce the noise of the individual species curves. The raw data neccessary to plot the curve is available from the supplemental material.

All datasets and analytic codes presented here are publicly available and open source at http://github.com/PalEON-Project/WitnessTrees, with the goal of enabling further analyses of ecological patterns across the region and the effects of post-settlement land-use on forest composition and structure. Data are also archived at the Long Term Ecological Research Network Data Portal (https://portal.lternet.edu/nis/home.jsp).

## Results

### Data Standardization

The original PLSS dataset contains 490,818 corner points (excluding line and meander points), with 166,607 points from Wisconsin, 231,083 points from Minnesota and 93,095 points from Michigan. Standardizing data and accounting for potential outliers, described above, removed 1.5% of points from the dataset, yielding a final total of 367,209 corner points.

Rasterizing the PLSS dataset to the Albers 64km^2^ grid produces 7,377 raster cells with data. Each cell contains between 0 and 101 corner points, with a mean of 66.5 (*σ* = 16.4) and a median of 72 corners (S3 File). Cells with a low number of points were mainly near water bodies or along political boundaries such as the Canadian/Minnesota border, or southern Minnesota and Wisconsin borders. Only 2.49% of cells have fewer than 10 points per cell.

Species assignments to genera were rarely problematic. Only 18 PLSS trees were assigned to the Unknown Tree category, representing less than 0.01% of all points. These unknown trees largely consisted of corner trees for which taxon could not be interpreted, but for which diameter and azimuth data was recorded. A further 0.011% of trees were assigned to the "Other hardwood" taxon (*e*.*g*., hawthorn, "may cherry", and "white thorn").

Maple has very high within-genera specificity for a number of assignments. A total of 78,478 trees are assigned to "Maple". Of these, surveyors do use common names that can be ascribed to the species level (e.g., *A*. *saccharum*, n = 56,331), but a large number of the remaining assignments are above the species level (n = 21,356). This lack of specificity for a large number of records causes challenges in using the species level data. A similar pattern is found for pine, where many individual trees (n = 125,639) can be identified to the level of species (*P*. *strobus*, n = 41,673; *P*. *banksiana*, n = 28,784; *P*. *resinosa*, n = 28,766), but there remains a large number of pine identified only at the genus level, or with unclear assignment (n = 17,606).

The data for ash includes both surveyor attributions to "brown ash" (likely a vernacular synonym for *Fraxinus nigra*) and black ash (n = 9,312), and white ash (n = 2,350), but again, also includes a large number of ash for which no distinction is made within the genera (n = 7,423).

These patterns are repeated throughout the data. For spruce this within-genera confusion is even higher, with 50,188 assignments to genera-level classes and only 17 to either black or white spruce.

### Spatial Patterns of Settlement-Era Forest Composition: Taxa and PFTs

#### Stem Density, Basal Area and Biomass

**Fig 2:**
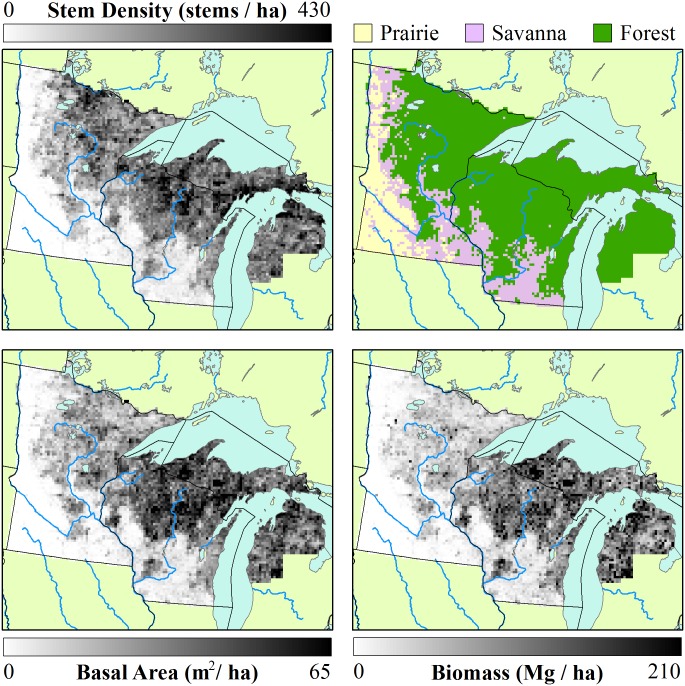
Structural characteristics of PLS era forests. Total stem density (a) in the Upper Midwest, along with forest type classification (b) based on PLSS data and the stem density thresholds defined by Anderson and Anderson [44] (Prairie: < 0.5 stems ha^-1^; Savanna: 0.5–47 stems ha^-1^; Forested: > 47 stems ha^-1^). Fine lines represent major rivers. To a first order, basal area (c) and biomass (d) show similar patterns to stem density (but see Fig 3).

The mean stem density for the region (Fig 2a) is 158 stems ha^-1^. Stem density exclusive of prairie is 177 stems ha^-1^ and is 221 stems ha^-1^ when both prairie and savanna are excluded. The 95th percentile range is 0–441 stems ha^-1^, and within-cell standard deviations between 0 and 447 stems ha^-1^. Basal area in the domain (Fig 2c) has a 95th percentile range between 0 and 66.1 m^2^ ha^-1^, a mean of 23.1 m^2^ ha^-1^, within-cell standard deviations range from 0 to 79.2 m^2^ ha^-1^. Biomass ranges from 0 to 218 Mg ha^-1^ (Fig 2d), with cell level standard deviations between 0 and 596 Mg ha^-1^. High within-cell standard deviations relative to mean values within cells for density, basal area and biomass indicate high levels of heterogeneity within cells, as expected for the PLSS data, given its dispersed sampling design.

In the PLSS data, stem density is lowest in the western and southwestern portions of the region, regions defined as prairie and savanna (Fig 2b). When the Anderson and Anderson [44] stem density thresholds (0.5–47 stems ha^-1^ for Savanna) are used, the extent of area classified as savanna is roughly equivalent to prior reconstructions [16,20,45] (Fig 2b). The highest stem densities occur in north-central Minnesota and in northeastern Wisconsin (Fig 2a), indicating younger forests and/or regions of lower forest productivity.

**Fig 3:**
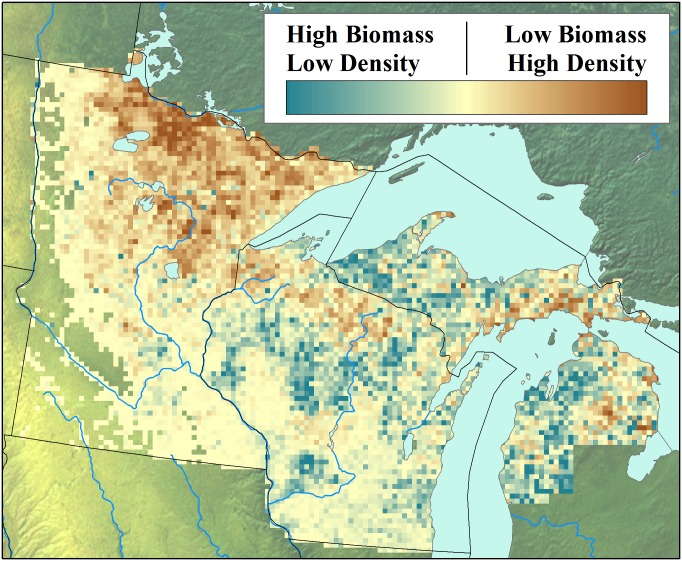
The ratio between biomass and stem density as an indicator of forest structure. Regions with high stem density to biomass ratios (brown) indicate dense stands of smaller trees, while regions with low stem density to biomass ratios (green) indicate larger trees with wider spacings.

Forest structure during the settlement era can be understood in part by examining the ratio of stem density to biomass, a measure that incorporates both tree size and stocking. Regions in northern Minnesota and northwestern Wisconsin had low biomass and high stem densities (Fig 3, brown). This indicates the presence of young, small-diameter, even-aged stands, possibly due to frequent stand-replacing fire disturbance in the pre-EuroAmerican period or to poor edaphic conditions. Fire-originated vegetation is supported by co-location with fire-prone landscapes in Wisconsin [94]. High-density, low-biomass regions also have shallower soils, colder climate, and resulting lower productivity. High-biomass values relative to stem density (Fig 3, green) are found in Michigan and southern Wisconsin. These regions have higher proportions of deciduous species, with higher tree diameters than in northern Minnesota.

#### Pre-Settlement Composition

**Fig 4:**
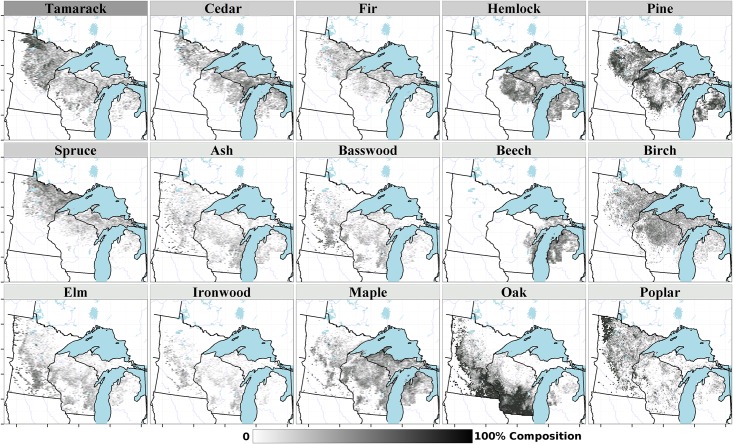
Forest composition (%, using stem density) for the 15 most abundant tree taxa in the PLSS. The scale is drawn using a square-root transform to emphasize low abundances. Shading of the bar above individual taxon maps indicates plant functional type assignments (dark gray: needleleaved deciduous; light gray: needleleaved evergreen; white: broadleaved deciduous).

Taxon composition (percent composition, based on stem density, unless otherwise indicated) within settlement-era forests is spatially structured along gradients from south to north (deciduous dominated to conifer dominated forests) and from east to west (mixed wood forests to open prairie) (Fig 4). Oak is dominant in the south of the region, with an average composition of 21%, however, that proportion drops to 9% when only forested cells (cells with stem density > 47 stems/ha) are considered, due to its prevalence as a monotypic dominant in the savanna and prairie. Pine distributions represent three dominant taxa, *Pinus strobus*, *Pinus resinosa* and *Pinus banksiana*. These three species have overlapping but ecologically dissimilar distributions, occurring in close proximity in some regions, such as central Wisconsin, and are typically associated with sandy soils with low water availability. Other taxa with high average composition in forested cells include maple (10%), birch (10%), tamarack (9%) and hemlock (8%).

Spruce in the PLSS represents two species (*Picea glauca*, *Picea mariana*) with overlapping distributions, but complex site preferences that vary in space. *P*. *glauca* is generally associated with dry upland to wet-mesic sites, while *P*. *mariana* is associated with hydric sites, but *P*. *mariana* also frequently occupies upland sites in northern Minnesota. Both cedar (*Thuja occidentalis*) and fir (*Abies balsamea*) are mono-specific genera in this region.

Northern hardwoods, such as yellow birch, sugar maple, and beech, are much less common in the lower peninsula of Michigan, and southern Wisconsin, except along Lake Michigan. Birch has extensive cover in the north, likely reflecting high pre-settlement proportions of yellow birch (*Betula alleghaniensis*) on mesic soils, and paper birch (*B*. *papyrifera*) on sandy fire-prone soils and in northern Minnesota (birch proportions reach upwards of 34.1% in northeastern Minnesota). Hardwoods in the southwest, such as oak, elm, ironwood and basswood, are most typically mono-specific groupings, with the exception of oak, which comprises 7 species (see S2 File). Hardwoods in the southwest are located primarily along the savanna and southern forest margins, or in the southern temperate deciduous forests. Finally, maple and poplar (aspen) have a broad regional distribution, occupying nearly the entire wooded domain. Poplar comprises four species in the region, while maple comprises five species (S2 File). Both hardwood classes, those limited to the southern portions of the region, and those with distributions across the domain, correspond to well-defined vegetation patterns for the region [16].

**Fig 5:**
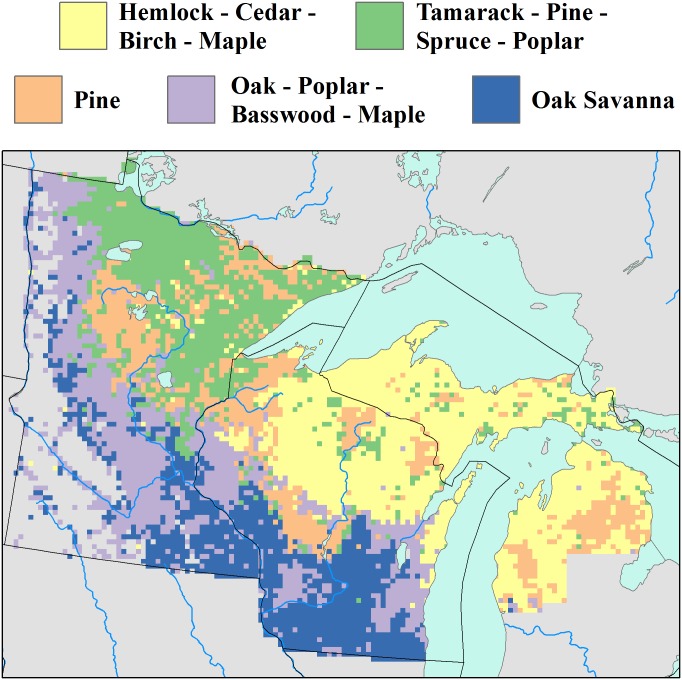
The five dominant forest types in the Upper Midwest as defined by k-medoid clustering. Forest types (from largest to smallest) include Hemlock-Cedar-Birch-Maple (yellow), Oak-Poplar-Basswood-Maple (light purple), Tamarack-Pine-Spruce-Poplar (light green), Oak Savanna (dark purple) and Pine (orange). These forest types represent meso-scale (64km^2^) forest associations, rather than local-scale associations.

Individual species distributions result in a mosaic of forest classes across the region (Fig 5). The dominant class is the Hemlock-Cedar-Birch-Maple assemblage in northern Wisconsin and upper Michigan (Fig 5, yellow). This mixedwood assemblage is interspersed by both Pine dominated landscapes (Fig 5, orange) and, to a lesser degree, the softwood assemblage Tamarack-Pine-Spruce-Poplar (Fig 5, green), which dominates in north-eastern Minnesota. The softwood assemblage is itself interspersed with Pine dominated landscapes, and grades into a mixed-hardwood assemblage of Oak-Poplar-Basswood-Maple (Fig 5, light purple) to the west. This mixed- softwood forest assemblage grades south into mono-specific Oak savanna (Fig 5, dark blue).

**Fig 6:**
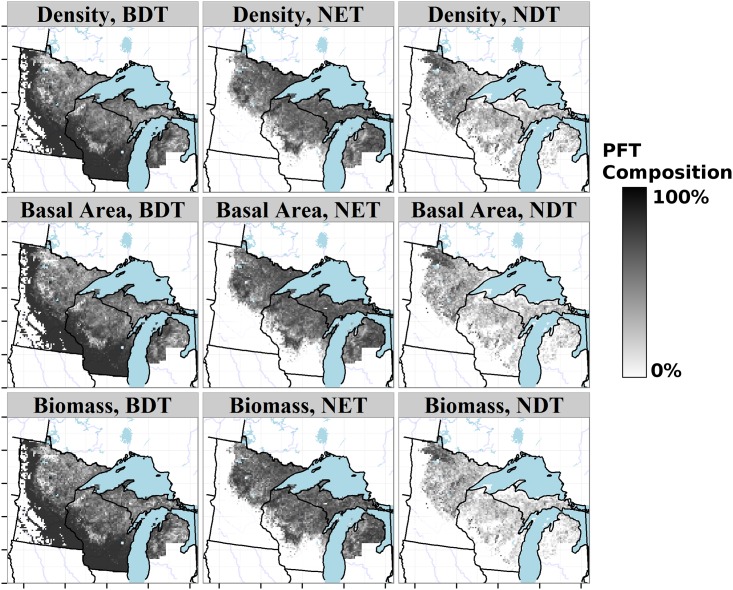
Proportional distribution of Plant Functional Types (PFTs) in the upper Midwest from PLSS data. Broadleaved deciduous tree (BDT), needleleaved deciduous tree (NDT), and needleleaved evergreen tree (NET) distributions are shown as proportions relative to total basal area, total biomass, and composition (Fig 2). The grassland PFT is mapped onto non-tree cells with the assumption that if trees were available surveyors would have sampled them.

The broad distributions of most plant functional types (Fig 6) results in patterns within individual PFTs that are dissimilar to the forest cover classes (Fig 5). Thus overlap among PFT distributions (Fig 6) emerges from the changing composition within the plant functional type from deciduous broadleaved species associated with the southern, deciduous dominated region, to broadleaved deciduous species associated with more northern regions in the upper Midwest.

### Structural Changes between PLSS and FIA Forests

**Fig 7:**
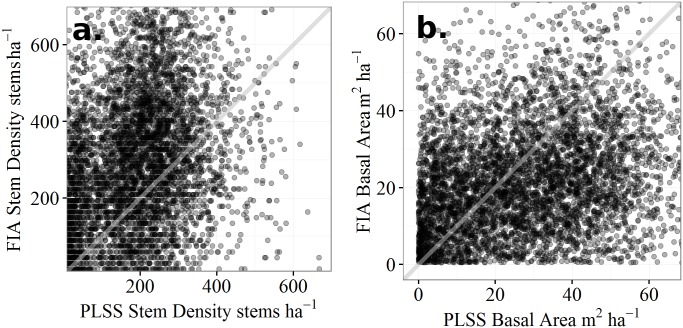
PLSS to FIA comparisons for stem density and basal area. Scatter plots of the relationship between (a) average stem density and (b) total basal area in the PLSS and FIA datasets. Stem density tends to be higher in the FIA, but total basal area tends to be higher in the PLSS. A 1:1 line has been added to the panels to indicate equality.

Comparing cell by cell in aggregate across the entire region, modern forests (FIA) have higher stem densities (+123 stems ha^-1^, *t*_1,5177_ = 43.9, *p* < 0.01) than PLSS forests, but lower basal areas (-5.67 m^2^ ha^-1^, *t*_1,5177_ = -19.3, *p* < 0.01) (Fig 7). We use only point pairs where both FIA and PLSS data occur since non-forested regions are excluded from the FIA and as such cannot be directly compared with PLSS estimates.

Every one of the five historical PLSS zones shows an increase in stem density (Table 2). The two forest types bordering the prairie, Oak Savanna and Oak-Poplar-Basswood-Maple both show increases in density and basal area that likely reflect, in part, the issues addressed earlier with respect to the sampling of forested plots in the FIA (over 10% cover). Density in the Oak Savanna in currently forested areas increases (Table 2). Oak-Poplar-Basswood-Maple forests were historically open forest and have seen a large increase in estimated FIA-era stem density, and a negligible increase in basal area (Table 2). The largest forest zone, Hemlock-Cedar-Birch-Maple shows the largest decline in basal area (Table 2).

**Table 2:** Mean cell-wise change in forest zone density and basal area since the PLSS for cells with coverage in both PLSS and FIA eras by forest class. All forest zones show increases in stem density since the PLSS era (positive values, historical values are included in parentheses). Oak Savanna and the Oak/Poplar/Basswood/Maple are the only zones with increasing basal area since the PLSS, all other zones show declines.

Forest Type	Area km^2^	*Δ* Stem Density stems ha^-1^	*Δ* Basal Area m^2^ ha^-1^
Hemlock/Cedar/Birch/Maple	1734	111.4 (280)	-18.3 (45.4)
Tamarack/Pine/Spruce/Poplar	1111	71.7 (224.4)	-4.8 (23.5)
Pine	966	167.6 (184.1)	-3.2 (27.1)
Oak/Poplar/Basswood/Maple	817	112.3 (93.5)	0.4 (14.8)
Oak Savanna	657	193.4 (29)	13.8 (4.7)

Across the forested region, pre-settlement forests have lower overall mean stem diameter than the FIA (*Δ*_*diam*_ = -4.93 cm, 95%CI from -27.8 to 8.04cm). This difference is strongest in the northwestern and western parts of the domain (on average modern forest data has 8.67 cm higher diameters), overlapping almost exactly with regions with low biomass to stem density ratios (Fig 3, brown regions). Conversely, regions with high biomass to stem density ratios, in north central Wisconsin, and the Upper and Lower Peninsulas of Michigan, had higher average diameters during the PLSS than in the FIA, by 3.79 cm. Hence, tree size has increased in the sub-boreal region and decreased in temperate mixedwood forests.

Differences between FIA and PLSS data in sampling design are unlikely to be a factor for most measures (see below); these differences are expected to affect how these datasets sample local- to landscape-scale heterogeneity, but should not affect the overall trends between datasets. Differences in variability introduce noise into the relationship, but given the large number of samples used here, the trends should be robust.

### Compositional Changes between PLSS and FIA Forests: Novel and Lost Forests

Both the PLS- and FIA-era compositional data show similar patterns of within-dataset dissimilarity, with the highest dissimilarities found in central Minnesota and northwestern Wisconsin. High within-PLSS dissimilarities are associated with high proportions of maple, birch and fir while high within-FIA dissimilarities are associated with high proportions of hemlock, cedar and fir. Dissimilarity values in the FIA dataset are less spatially structured than in the PLSS. Moran's I for dissimilarities within the FIA (*I*_*FIA*_ = 0.17, p < 0.001) are lower than the dissimilarities within the PLSS (*I*_*PLSS*_ = 0.495, p < 0.001), suggesting lower spatial autocorrelation in the FIA dataset. Cells with identical pairs represent 5.56% of the PLSS cells and 8.43% of FIA cells. Identical cells in the PLSS are largely located along the southern margin and most (69.9%) are composed entirely of oak. Cells in the FIA with identical compositional neighbors are composed of either pure oak (16.3%), pure poplar (24%) or pure ash (11%).

There is a small but significant positive relationship (*F*_1,5094_ = 13.5, p < 0.001) between the number of FIA plots and within-FIA dissimilarity once the spatial relationship has been accounted for. The relationship accounts for < 1% of total variance and estimates an increase of *Δ*_*d*_ = 0.00176 for every FIA plot within a cell. This increase represents only 0.306% of the total range of dissimilarity values for the FIA data. There is a gradient of species richness that is co-linear with the number of FIA plots within a cell, where the number of plots increases from open forest in the south-west to closed canopy, mixed forest in the Upper Peninsula of Michigan. There is also a significant positive relationship between taxon richness and plot number, which complicates the analysis. Hence, differences in within- and between-cell variability between the PLSS and FIA datasets seem to have only a minor effect on these regional-scale dissimilarity analyses.

**Fig 8:**
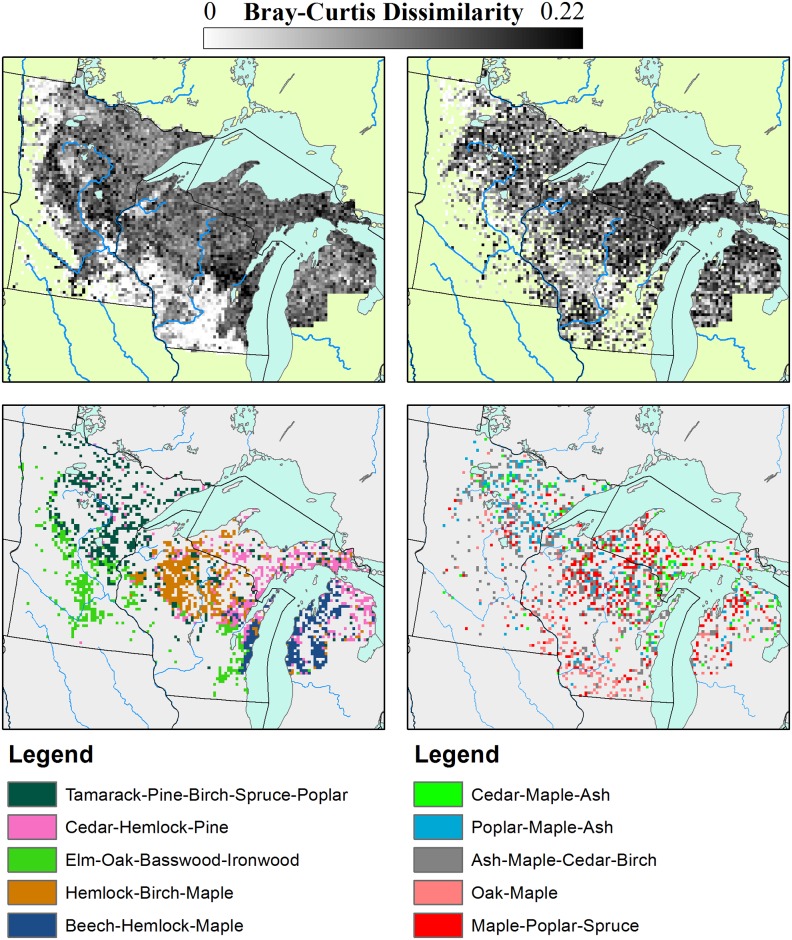
Minimum dissimilarity maps. Distributions of minimum (within dataset) dissimilarities during the PLSS (a) and FIA (b) show spatially structured patterns of dissimilarity, with stronger spatial coherence for the PLS. Lost forests (c) show strong compositional and spatial coherence, and have more taxa with percent composition > 10% than within Novel forests during the FIA era (d). The lost Elm-Oak-Basswood-Ironwood zone lies south of the Tension Zone [16,72], while the other zones lie largely north or east of the Tension Zone. The spatial structure of the novel forests is less well defined.

We define novel communities as those whose nearest neighbor is beyond the 95%ile for dissimilarities within a particular dataset. In the PLSS dataset, forests that have no modern analogs are defined as "lost forests", while forest types in the FIA with no past analogs are defined as "novel forests". More than 28% of PLSS sites have no analog in the FIA dataset ('lost forests'; PLS-FIA dissimilarity, Fig 8c), while 28% of FIA sites have no analog in the PLSS data ('novel forests'; FIA-PLSS dissimilarity, Fig 8d).

Lost forests are drawn from across the domain, and show strong ecological and spatial coherence (Fig 8c). The lost forests fall into five classes: Tamarack-Pine-Birch-Spruce-Poplar accounts for 26.9% of all lost forests and 8.2% of the total region. This forest type is largely found in north eastern Minnesota, extending southward to central Minnesota, into Wisconsin and along the Upper Peninsula of Michigan, as well as in scattered locations on the Lower Peninsula of Michigan (Fig 8c). This forest likely represents a mesic to hydric forest assemblage, particularly further eastward. Modern forests spatially overlapping this lost type are largely composed of poplar (${\bar{x}}_{FIA}$ = 17.4%), ash (${\bar{x}}_{FIA}$ = 16.6%) and oak (${\bar{x}}_{FIA}$ = 16.4%). Tamarack in these forests has declined significantly, from 19.1% to only 3.48% in the FIA, while poplar has increased from 9.25% to 17.4%, resulting in forests that look more like early successional forests.

Cedar/Juniper-Pine-Maple-Hemlock accounts for 20.9% of all lost forests and 6.39% of the total region. This forest type is found largely in northeastern Wisconsin, and the Upper and Lower Peninsulas of Michigan. This lost forest type has been predominantly replaced by maple, poplar, and pine, retaining relatively high levels of cedar (${\bar{x}}_{PLS}$ = 21.4%; ${\bar{x}}_{FIA}$ = 17.9%). The loss of hemlock is widespread across the region, but particularly within this forest type, declining to only 1.08% from a pre-settlement average of 11.7%.

Elm-Oak-Basswood-Ironwood accounts for 17.2% of all lost forests and 5.25% of the total region. The region is centered largely within savanna and prairie-forest margins, both in south-central Minnesota and in eastern Wisconsin, but, is largely absent from savanna in the Driftless area of southwestern Wisconsin. In particular, much of this zone lies in the Big Woods region of Minnesota [95,96]. These forests were historically elm dominated (${\bar{x}}_{PLS}$ = 16.4%), not oak dominated savanna, as elsewhere (particularly in the Driftless). Modern forests replacing these stands are dominated by oak and ash, with strong components of maple, and basswood. Elm has declined strongly in modern forests (${\bar{x}}_{FIA}$ = 5.97%), possibly in part due to Dutch Elm Disease and land-use. The increase in ash in these forests is substantial, from $\;{\bar{x}}_{PLS}$ = 6.52% to ${\bar{x}}_{FIA}$ = 15.1%.

Hemlock-Birch-Maple accounts for 20.1% of all lost forests and 6.13% of the total region. This forest type, dominant in north central Wisconsin, was dominated by hemlock ($\bar{\_}x_{PLS}$ = 26.9%) and what was likely late seral yellow birch (${\bar{x}}_{PLS}$ = 22.2%), replaced largely by maple (from ${\bar{x}}_{PLS}$ = 12.1% to ${\bar{x}}_{FIA}$ = 29.3%). Poplar increases from 1.06% to 12.7% in the FIA, again indicating a shift to earlier seral forests in the FIA. Hemlock is almost entirely lost from the forests, declining from 26.9% to 4.23% in the FIA.

Lastly, Beech-Hemlock-Maple accounts for 14.9% of all lost forests and 4.55% of the total region. This forest type is found exclusively on the central, western shore of Lake Michigan and in the Lower Peninsula, in part due to the limited geographic range of Beech in the PLSS dataset (Fig 4). Beech is almost entirely excluded from the modern forests in this region, declining from $\;{\bar{x}}_{PLS}$ = 35.6% to ${\bar{x}}_{FIA}$ = 3.94%. Pine in the region increases from 5.89% to 5.79%, while maple, the dominant taxa in the modern forests, increases from 17.4 to 27.2%.

On average lost forests contain higher proportions of beech (r = 0.278), hemlock (r = 0.233), birch (r = 0.22) and ironwood (r = 0.199) than the average PLSS forest, and lower proportions of oak (r = -0.311), poplar (r = -0.149), and pine (r = -0.126).

The distribution of novel ecosystems (Fig 8d) is spatially diffuse relative to the lost forest of the PLSS and the forest types tend to have fewer co-dominant taxa. FIA novel forest types also have a more uneven distribution in proportion than the PLSS lost forests and much weaker relationships with individual taxa, with no taxon association higher than 0.141 (ash) or lower than -0.233 (tamarack). This suggests that the loss of particular forest types associated with post-settlement land-use was concentrated in mesic deciduous forests and the ecotonal transition between southern and northern hardwood forests, while novel forests were more dispersed, resulting from an overall decline in seral age.

By far the largest novel forest type is Ash-Maple-Cedar/Juniper-Birch, which accounts for 35.5% of all novel forests and 9.07% of the total region. As with all novel forest types, this forest type is broadly distributed across the region. This forest type is associated with co-dominant maple (${\bar{x}}_{FIA}$ = 23%) and ash (${\bar{x}}_{FIA}$ = 22%). Hemlock has declined significantly across this forest type, from ${\bar{x}}_{PLS}$ = 12.3% to ${\bar{x}}_{FIA}$ = 3.48%, as has tamarack (from ${\bar{x}}_{PLS}$ = 10.2% to ${\bar{x}}_{FIA}$ = 2.71%) while ash has increased from, from ${\bar{x}}_{PLS}$ = 3.75% to ${\bar{x}}_{FIA}$ = 22.9%

Maple-Poplar-Spruce, accounts for 22.9% of all novel forests and 5.85% of the total region. The broad distribution of these novel forests makes assigning a past forest type more difficult than for the PLSS lost forests, the distribution is coincident with two classes of pre-settlement forest, Oak Savanna and Hemlock-Cedar-Birch-Maple (Figs 5 and 8). Here maple has increased significantly, from ${\bar{x}}_{PLS}$ = 10.9% to ${\bar{x}}_{FIA}$ = 15.7%. This gain comes at the expense of hemlock and birch, both of which decline by over 10% from the PLS to the modern era.

Poplar-Maple-Ash forest accounts for 15.6% of all novel forests and 3.97% of the total region. This forest type is again broadly distributed across the region, representing a homogenous, early seral forest type, likely associated with more mesic sites. Oak-Maple forest accounts for 14.4% of all novel forests and 3.69% of the total region. The last novel assemblage Poplar-Maple-Ash accounts for 15.6% of all novel forests and 3.97% of the total region. Among all novel assemblages we see maple and ash as significant components (increasing on average by >10% across the novel forest zones), replacing hemlock, tamarch and birch, which decrease by > 6% within the novel forest zones.

### Spatial Correlates of Novelty

Modern compositional dissimilarity from the PLSS data is related to distance from 'remnant' forest. The dissimilarity quantile of FIA-PLSS distances increases with increasing distance to remnant cells, and this relationship is robust to higher thresholds for defining remnant forest classification, up to the 90%ile of within-PLSS near neighbor dissimilarities. Using the 25%ile for within-PLSS dissimilarity, approximately 69% of FIA cells can be classed as 'remnant' forest. The mean distance to remnant forests for cells with dissimilarities above the 25%ile is 15.2 km, higher than the mean of ~9.6km expected if each 8x8km cell had at least one adjacent 'remnant' cell.

**Fig 9:**
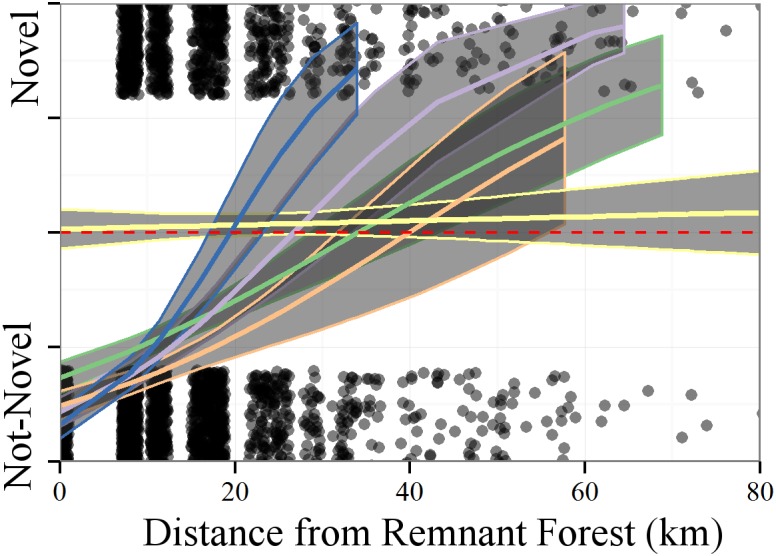
The model relating the probability of novel forests to spatial distance from remnant forest. Here the 25%ile is used to indicate remnant forest, and the 95%ile is defined as novelty. Binomial regression predicts the probability of novel forest, the red dashed line indicates a response greater than 0.5. The curves represent the relationship between spatial distance and dissimilarity for each of the five major historic forest types (Fig 5) defined here as Oak Savanna (blue), Oak-Poplar-Basswood-Maple (light purple), Tamarack-Pine-Spruce-Poplar (green), Hemlock-Cedar-Birch-Maple (yellow) and Pine (orange). Points are jittered to improve display. Points at 1 are cells whose dissimilarity is greater than the 95th %ile of dissimilarities within the PLSS, here considered novel forest.

The GLM shows that distance from remnant forests in the FIA is significantly related to the probability of a cell being novel (*χ*_*1*,*4*_ = 623, p < 0.001). The mean distance to novel forest varies by pre-settlement forest class (Figs 5 and 9), but is between approximately 20 and 60km for the four forest types examined here (Fig 9), while the null model would predict a distance of 10 – 20km to novelty from remnant cells if dissimilarities were distributed randomly on the landscape (Table 3). Novel forests are generally further from remnant patches than expected in the null model, regardless of forest type, but the distance to novelty is greater for modern forests that are, generally, more similar to their PLSS state (pine and tamarack dominated forests), and closer for forests that are more dissimilar.

**Table 3:** Spatial distance to novelty—modeled as a binomial—from remnant forests (forests within the first 25th percentile of nearest neighbor distances). The null model uses permutation (n = 100) where quantiles are resampled without replacement.

Zone	Min	Max	Min (Null)	Max (Null)
Tamarack-Pine-Spruce-Poplar	23	31	10.88	13.57
Oak-Poplar-Basswood-Maple	20	26	17.26	17.26
Pine	31	43	9.34	11
Hemlock-Cedar-Birch-Maple	22	36	11.87	14.06
Oak Savanna	18	25	11.97	15.84

**Fig 10:**
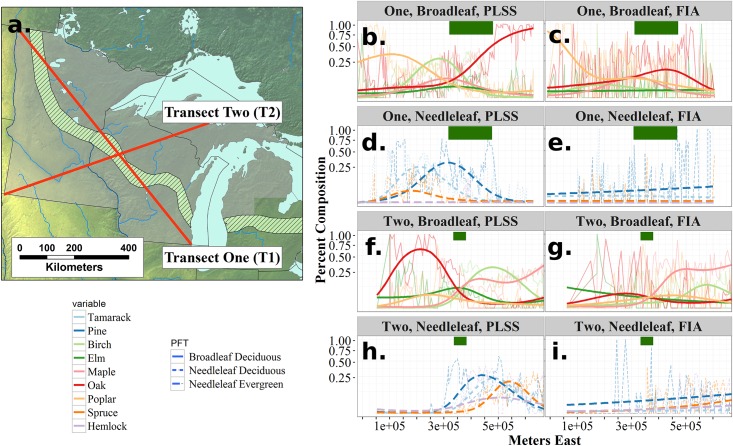
Transects (a) across the region show clear changes in the ecotonal strength. Transect One shows shifts in broadleaved taxon distributions from the PLSS to FIA (b and c) and in needleleaved distributions (d and e). Transect Two broadleaf (f and g) and needleleaf (h and i) taxa show shifts that again appear to represent regional scale homogenization. Ecotones in the pre-settlement era were stronger in the past than they are in the present. Fitted curves represent smoothed estimates across the transects using Generalized Additive Models using a beta family.

The Hemlock-Cedar-Birch-Maple forest class (Figs 5 & 10b, yellow), appearing as a flat line, predicting novel forest continuously, from distance 0. This is due, in part, to the very small proportion of Hemlock-Cedar-Birch-Maple cells that are considered residual (only 63 of 1780 FIA cells within the historical zone are considered remnant) and the very high proportion of novel cells in the zone (923 of 1780 cells, or 52% of all cells).

Distance to novel forests within the Oak Savanna have a confidence interval that overlaps the interval predicted from the null model (Table 3). Northern softwood forests (Tamarack-Pine-Spruce-Poplar, Fig 5, light green) are considered novel at between 29 and 43km, northern Oak forests (Oak-Poplar-Basswood-Maple; Fig 5, light purple) are predicted to be novel at 23–33 km, slightly higher than the 14 – 19km predicted by the null model. Pine forests (Fig 5, orange) are predicted to be novel at a distance three times further than expected by the null, at 32 – 56km (Table 3).

### Compositional Changes between PLSS and FIA Forests: Ecotone Structure

To understand how the ecotonal structure has been transformed by post-settlement land-use, we constructed two transects of the FIA and PLSS data (Fig 10a), and fitted GAM models to genus abundances along these transects. Transect One (T1) runs from northern prairie (in northern Minnesota) to southern deciduous savanna in southeastern Wisconsin (left to right in Fig 10c–10f), while Transect Two (T2) runs from southern prairie in southwestern Minnesota to northern mixedwood forest in the Upper Peninsula of Michigan (left to right in Fig 10g–10j). In general, these transect analyses show: 1) significant differences in ecotonal structure between the present and pre-settlement, and 2) steeper ecotones in the past and more diffuse ecotones today.

For T1, GAM models show significant differences (using AIC) between time periods in curves for all broadleaved taxa (Fig 10b & 10c) and for al needleleaved taxa (Fig 10d and 10e). The PLSS curves show a transition in the northwest from poplar dominated open forest to a needleleaved forest composed of pine, spruce and tamarack. Tamarack and poplar proportions decline gradually from the east, being replaced first by pine, then briefly by maple and birch, and then, ultimately by oak as the transect grades into oak savanna. In the FIA dataset poplar in the northwest grades into mixedwood forests. While the PLSS transect shows distinct vegetation types in the central part of the transect, the FIA shows relatively constant proportions of oak, pine, spruce, poplar and maple before pine, oak and elm increase in the southeastern portions of the transect.

The second transect (T2) shows a similar pattern, with well-defined ecotones in the pre-settlement period (Fig 10f and 10h). Although a sharp increase in poplar appears just east of the Tension Zone in (Fig 10g), there are no major changes in any other taxon (Fig 10g and 10i). Oak forest, with a component of elm and poplar in the southwest grades slowly to a rapid transition zone where pine, elm, maple (first), then rapidly birch, hemlock and tamarack, and later, spruce, increase. This region, the Tension Zone, extends from 3 x 10^5^ to 4.5x10^5^ meters East, eventually becoming a forest that shows co-dominance between birch, pine, maple, spruce and tamarack, likely reflecting some local variability as a result of topographic and hydrological factors. Missing data at the beginning of the FIA transect reflects a lack of FIA plots in unforested regions in the west

Contemporary forests show broader homogenization and increased heterogeneity (evidenced by the lower within-FIA Moran's I estimates for near-neighbor distances) at a local scale in the region. Homogenization is evident across T1, where Bray-Curtis dissimilarity between adjacent cells declines from the PLSS to the FIA (*Δ*_*beta*_ = -0.22, *t*_113_ = -7.93, p<0.001), mirroring declines in the Pine Barrens between the 1950s and the present [28]. The PLSS shows strong differentiation in the central region of T2 where maple-pine-oak shifts to pine-poplar-birch forest (Fig 10d). This sharp ecotone is not apparent in the FIA data, which shows gradual and blurred changes in species composition across the ecotone (Fig 10i). *β*-diversity along T2 is lower in the FIA than in the PLSS (*Δ*_*beta*_ = -0.19, *t*_65_ = -7.34, p < 0.01), indicating higher heterogeneity in the PLSS data at the 64 km^2^ meso-scale.

Across the entire domain, *β* diversity is lower in the FIA than in the PLSS (*Δ*_*β*_ = -0.172, *t*_1.3*e*7_ = 2480, p <0.001), lending support to the hypothesis of overall homogenization. Differences in sampling design between PLSS and FIA data cannot explain this homogenzation, since its effect would have been expected to increase *β*-diversity along linear transects and at larger spatial scales.

## Discussion

The records of the Public Land Survey System (PLSS) provide broad spatial coverage for the Upper Midwestern United States and elsewhere at a time immediately prior to widespread land use change. Many of the forests of the PLSS are no longer represented on the landscape. We identify five key forest-types that have vanished at the 64 km^2^ mesoscale, and five new forest types that have been gained. The joint controls of broad-scale climatic structuring and local hydrology on forest composition and density can be seen in the pre-settlement forests. For example, along the Minnesota River in south-western Minnesota a corridor of savanna was sustained in a region occupied by prairie (Fig 2b). Composition gradients in the FIA are weaker now than in the past (Fig 10), with clear signs of increased homogenization at local and regional scales and decreased spatial structure among forest-types (Fig 8).

The loss of ecotones in the upper Midwestern United States suggests that ability to predict abiotic controls on species distributions at the landscape scale may be weaker than in the past, reducing the apparent influence of climatic or edaphic factors, and increasing the relative influence of recent land-use history, a factor that is often not considered [30].

Recent land-use history and historical vegetation cover play a large role in recovery from the large-scale disturbance following EuroAmerican settlement. Our results show decreased *β* diversity along regional transects (shown in Fig 10), which indicates homogenization at meso-scales of 100s of km^2^, while the overall reduction in Moran's I for dissimilarity in the FIA indicates a regional reduction in heterogeneity on the scale of 1000s of km^2^. The selective loss or weakening of major vegetation ecotones, particularly in central Wisconsin, and the development of novel species assemblages across the region further suggests that modern correlational studies, examining regional relationships between species and climate (for example) may fail to capture the full range of edaphic controls on species distributions. These changes are the result of land-use, both agricultural and logging, but affect forests in contrasting ways across the domain. Maple has become one of the most dominant taxa across the region, largely red maple (*Acer rubrum*), while in northern Minnesota, species shifts have reflected increases in poplar and pine, while in south central and eastern Wisconsin hemlock has been lost almost completely.

Recent work in eastern North America suggests the utility of including spatial structure in species distribution models to improve predictive ability [97]. Accounting for spatial structure may improve models by capturing missing covariates within species distribution models [97], but if recent land-use history has strongly shaped species distributions, or co-occurrence, then the spatial effect is likely to be non-stationary at longer temporal scales. Observations at longer time-scales, and multiple baselines from which to build distributional models are critical to avoid conflating recent land-use effects with the long-term ecological processes structuring the landscape [32,98].

Anthropogenic shifts in forest composition over decades and centuries seen here and elsewhere [2,59] are embedded within a set of interacting systems that operate on multiple scales of space and time [99]. Combining regional historical baselines, long-term ecological studies and high frequency analyses can reveal complex responses to climate change at local and regional scales [100]. Estimates of pre-settlement forest composition and structure are critical to understanding the processes that govern forest dynamics because they represent a snapshot of the landscape prior to major EuroAmerican land-use conversion [42,62]. Pre-settlement vegetation provides an opportunity to test forest-climate relationships prior to land-use conversion and to test dynamic vegetation models in a data assimilation framework [101]. For these reason, the widespread loss of regional forest associations common in the PLSS (Fig 8d), and the rapid rise of novel forest assemblages (Fig 8e) have important implications for our ability to understand ecological responses to changing climate. The loss of historical forest types implies the modern understanding of forest cover, climate relationships, realized and potential niches and species associations may be biased in this region, given that 28% of the total regional cover is novel relative to forests only two centuries ago and that forests prior to EuroAmerican change were also undergoing shifts in composition on relatively short time-scales [95,96]

Beyond shifts in composition at a meso-scale, the broader shifts in ecotones can strongly impact models of species responses and co-occurrence on the landscape. For example, the heterogeneity, distribution, and control of savanna-forest boundaries [102] is of particular interest to ecologists and modelers given the ecological implications of current woody encroachment on savanna ecosystems [103]. Declines in landscape heterogeneity may also strongly affect ecosystem models, and predictions of future change. Our data show higher levels of vegetation heterogeneity at mesoscales during the pre-settlement era, and greater fine scaled turnover along transects. Lower *β* diversity shown here and elsewhere [28] indicate increasing homogeneity at a very large spatial scale, and the loss of resolution along major historical ecotones.

This study also points to the need for a deeper understanding of some of the landscape- and regional-scale drivers of novelty, given the likely role for climatic and land-use change (including land abandonment) to continue to drive ecological novelty [35,104]. In particular the role of regional species pools and remnant patches of forest in driving or mitigating compositional novelty. This work shows that the baseline forest type, and its structure on the landscape moderates the degree to which landscape scale patterns can drive compositional novelty. To some degree relationships between compositional novelty and distance from remnant patches may be dependent on the size or diversity of the species pool and the sensitivity of dissimilarity metrics to *β* diversity [105]. Our results indicate that diversity alone cannot be the driving factor in determining post-settlement dissimilarity since all forest classes show this pattern of change.

Both Pine and the Oak-Poplar-Basswood-Maple forest types are the most fragmented across the region. There is strong evidence that, in some locations, pine forests have persisted over very long timescales in the region [106], although there is also evidence, in other regions, that these states may shift strongly in response to interactions between landscape level processes such as fire and geophysical features [107]. Thus complex interactions between landscape-sc ale processes, including fire and land-use change, or geophysical features, and the species assemblages themselves, point to the difficulty in making simplifying assumptions about species assemblages. Caution in simplifying species assignments is necessary since this region is dominated by forests that respond very differently to the settlement-era (and pre-settlement) disturbance, even though they are composed of different species within the same genera or plant functional type. Recent ecosystem model benchmarking using pre-settlement vegetation has shown significant mismatch between climate representations of plant functional types across a range of ecosystem models [21]. In the model intercomparison, no ecosystem model accurately represented the true climate space of plant functional types in the northeastern United States, indicating simplifying assumptions within dynamic models of vegetation fail to accurately represent the complexity of vegetation in this region with respect to climate [21].

The analysis relating to the distance-to-novel forests (Fig 9) points to the possibility that landscape-scale restoration has high likelihood of success if local-scale restoration focuses on sites where restoration potential is high, as suggested for Hemlock-Cedar-Birch-Maple forests in northern Wisconsin [45]. If some of the novelty is driven by depauparate species pools beyond certain threshold distances from remnant forests then it should also be possible to restore these forest at a regional scale through the translocation of key species [108]. This work is supported by a number of other studies at smaller scales [109–111]. For example, the presence of white pine in mesic sites during the PLSS era has been attributed to its presence as a seed source on marginal sites at scales of hundreds of meters [112]. Simulations show that seed-source distribution can affect community composition over hundreds of years at large spatial scales in a region spatially coincident with this current study [113]. Thus, land-use change has significantly altered the landscape, both by "resetting" the successional clock, but also, because of the extent of change, by impacting the regional species pool and seed source for re-establishing forests that are compositionally similar to pre-settlement forests.

Methodological advances of the current work include 1) the systematic standardization of PLSS data to enable mapping at broad spatial extent and high spatial resolution, 2) the use of spatially varying correction factors to accommodate variations among surveyors in sampling design, and 3) parallel analysis of FIA datasets to enable comparisons of forest composition and structure between contemporary and historical time periods. This approach is currently being extended to TPS and PLSS datasets across the north-central and northeastern US, with the goal of providing consistent reconstructions of forest composition and structure for northeastern US forests at the time of EuroAmerican forests.

Our results support the consensus that robust estimates of pre-settlement forest composition and structure can be obtained from PLSS data [47,48,65,114,115]. Patterns of density, basal area and biomass are roughly equivalent to previous estimates [17,26], but show variability across the region, largely structured by historical vegetation type (Table 3). Our results for stem density are lower than those estimated by Hanberrry *et al*. [27] for eastern Minnesota, but density and basal area are similar to those in the northern Lower Peninsula of Michigan [19] and biomass estimates are in line with estimates of aboveground carbon for Wisconsin [17].

These maps of settlement-era forest composition and structure can provide a useful calibration dataset for pollen-based vegetation reconstructions for time periods predating the historical record [50]. Many papers have used calibration datasets comprised of modern pollen samples to build transfer functions for inferring past climates and vegetation from fossil pollen records [116–119]. However, modern pollen datasets are potentially confounded by recent land-use, which can alter paleoclimatic reconstructions using pollen data [118]. By linking pollen and vegetation at modern and historical periods we develop capacity to provide compositional datasets at broader spatio-temporal scales, providing more data for model validation and improvement. Ultimately, it should be possible to assimilate these empirical reconstructions of past vegetation with dynamic vegetation models in order to infer forest composition and biomass during past climate changes. Data assimilation, however, requires assessment of observational and model uncertainty in the data sources used for data assimilation. Spatio-temporal models of uncertainty have been developed for the compositional data [73].

Ultimately the pre-settlement vegetation data present an opportunity to develop and refine statistical and mechanistic models of terrestrial vegetation that can take multiple structural and compositional forest attributes into account. The future development of uncertainty estimates for the data remains an opportunity that can help integrate pre-settlement estimates of composition and structure into a data assimilation framework to build more complete and more accurate reconstructions of past vegetation dynamics, and to help improve predictions of future vegetation under global change scenarios.

## Supporting Information

S1 File Zip file for all data processing. (DOCX)

S2 File Zip file with taxon assignments for PLSS surveyor records. (ZIP)

S3 File Zip archive with base raster and gridded data output as csv files. (ZIP)

S4 File Zip archive with correction factors for PLSS survey data. (ZIP)
