# Supplementary material for: Novel and Lost Forests in the Upper Midwestern United States, from New Estimates of Settlement-Era Composition, Stem Density, and Biomass
Source: PLoS One. 2016 Dec 9;11(12):e0151935. doi: 10.1371/journal.pone.0151935 (PMC5147790; doi:10.1371/journal.pone.0151935)
Supplement: S1 File — (DOCX) [file pone.0151935.s001.docx]

S1 File

Data have been uploaded to Fighsare at https://dx.doi.org/10.6084/m9.figshare.4024860.v1
